# Success in Life

**Published:** 1871-08

**Authors:** 


### SUCCESS IN LIFE,

By which is generally meant the acquisition of wealth, but may reasonably be extended to the attainment of places of honor, trust, influence, or power of doing good, is oftenest the result of three mental characteristics: observation, judgment, perseverance. The first gives the exact knowledge of the facts; the second directs to the best method of using them ; the third implies a persistent use of them, a concentration of all the energies of a man's nature upon this one occupation, calling, or trade, or whatever it may be.

The beginning of every man's fortune is in the exercise of an accurate, critical, and comprehensive observation. A superficial observation is an almost universal defect in the American character. Of fifty persons who witness the marvel of a printing-press, turning out — printing and folding — ten or twenty thousand newspapers in an hour without the touch of a human hand, there might be one or two who would be anxious to look closely into the mechanical structure, and continue to look and examine until there was a perfect understanding of the mode of its working ; the great majority being satisfied with expressing some word of gratification or astonishment, not retaining enough in the mind to describe a single piece of the machinery.

It is a frequent remark about men who have accumulated large fortunes by their own individual exertions, that they have not two ideas beyond their own particular calling, may not know even who is the governor of the State, or whether General Jackson is alive or not; these men had but little intelligence, but they concentrated their whole being, every power of body, every faculty of mind, on the one great work of their lives.

Newton saw the apple fall; his judgment led to the conjecture of the causes. Not one in a hundred perhaps would inquire what made it fall, and if the same thing made everything else fall.

Furguson saw a watch ticking and moving, he was struck with wonder, but he was not satisfied there; he wanted to know what made it tick and move, and kept up the investigation until he found it out; and this led to greater things, which made him immortal. The great road to super-eminent success in life is accurate, critical observation, a comprehensive judgment, a persistent action, — that is, an indomitable perseverance.

